# Gastrostomy for Occlusion of Œsophagus

**Published:** 1889-08

**Authors:** 


### EDITORIALS AND MISCELLANEOUS.

### GASTROSTOMY FOR OCCLUSION OF ŒSOPHAGUS.

A Surgical case of more than ordinary interest occurred in the practice of Dr. J. McFadden Gaston recently, consisting in stenosis of the œsophagus, of long standing, and ultimately terminating in complete occlusion of the canal, just above the diaphragm.

The patient was a white man 35 years old, under the care of Dr. A. C. Moreland, who requested Dr. Gaston to undertake the operation of gastrostomy, after attempting rectal alimentation for two weeks; and the case was accordingly turned over to him on July 8th for preparatory treatment. This consisted in the addition of half an ounce of tincture of Peruvian bark to the nutritive anemate every four hours, until July 13th, when the operation was performed in the following manner:

An incision, four inches long, extending from the median line obliquely across, one inch below the cartilages of the ribs and the left side, was carried through all the structures into the peritoneal cavity. Four fingers of the left hand were introduced, and after considerable difficulty, owing to the contracted state of the coats of the stomach, the organ was brought into the external opening. Two strong silk ligatures were passed at a distance of an inch and a half through the walls of the anterior position of the large curvature of the stomach, and secured by an assistant, wnile an incision of one inch was made in the intervening tissue, entering the cavity. The fore finger of the operator's left hand was then pressed up to the cardiac orifice, and with this as a guide, an olive pointed elastic probang, was introduced and carried up through the constricted part, about two inches above the cardiac opening, requiring only moderate force in the passage of a bougie being half an inch in diameter. The edges of the parietal peritoneum were closed with continuous catgut suture to the point, when the incised walls of the stomach were drawn into the external wound, and then secured around this by stitches, including only the servus and muscular layers, at a distance of half an inch from the edges of the stomach. A deep interrupted silk suture was used to close the painted wound up to the included margins of the gastric walls; and these were attached to the edges of the parietal incision, by first passing interrupted stitches of silk and catgut alternately through both, which were left for the time united. A silver canula of half inch diameter, ¾ in. long, having flanges at both ends was now introduced into the opening, and the stitches tightened and tied around the entire margin of the gastric aperture so as to fix the canula in its position. An india rubber stopper closed the tube, so as to be removed at will, for the introduction of nourishment. The tegumentary union was covered with iodoform, with antiseptic cotton, and a sheet of protective oil cloth outside, the whole being secured by turns of a broad roller bandage around the body.

After recovering from the effects of the anæsthetic (A. C. E. mixture) the patient took a sip of water, declaring that it went into the stomach, and afterwards used milk and lime water, or milk punch repeatedly in small quantities. But this was followed on the second and third days by regurgitation.

On the evening of the 16th milk was thrown into the stomach through the canula, but returned immediately by the constricting of the stomach.

From the outset, after the operation, enemata of beef soup, corn meal gruel and tincture of Peruvian bark were kept up every four hours, with a view to relieve the stomach; and after this unfavorable experiment with feeding by the mouth or the canula, the necessity of continuing this mode of alimentation was very apparent.

The temperature and pulse beat had not gone much beyond the normal prior to the morning of the 17th, when all had changed for the worse, with an accelerated pulse and temperature of 103¼ deg. F. It had been necessary to draw ofl the urine with the catheter frequently, while at times it was passed voluntarily, and the quantity on this occasion exceeded half a pint, showing that the secretion or excretion was progressing regularly.

There was no distension of the abdomen indicating local peritonitis, and the patient swallowed peptonized milk with lime water without greater difficulty than on previous occasions. But the mind was somewhat impaired, and the vital powers evidently giving way, so that it gave no surprise to learn that he died at 2 p. m. of that day, being the fourth after the gastrostomy was done.

An autopsy, four hours after death, showed the external wound united, with the exception of a single point in the peritoneum, near the border of the gastric attachment, while the union between the gastric and parietal walls was complete. The stricture in the œsophagus embraced all its coats for an extent of 2 inches, and was much thickened and indurated, with a tendency to disintegration of structure immediately below this hard mass.

The indications of scirrhus degeneration are such that there was no prospect of final relief, even should the patient have survived the measure of temporary alimentation. The specimen will be subjected to examination by the microscope, for decision as to the character of the disease.

Dr. Gaston was assisted in the operation by Drs. W. D. & B. W Bizzell, Dr. Crichton, Dr. C. D. Roy and Dr. Sims. Sickness prevented the presence of Dr. Moreland.

It should be stated that this operation was proposed to the patient more than a month previous to its performance, while he was able to swallow semi-fluid nourishment of milk and soft eggs, and consequently in a much more favorable condition for recovery. But it was declined, and he persisted in refusing to have it done, with the hope that the occlusion might yield, until his vital force was too much exhausted, for recuperation.

The unfavorable results of gastrostomy in chronic stenosis and ultimate occlusion of the œsophagus should prove a warning to patients and surgeons to resort to the operation at so early a period as may prevent the untoward consequences of inanition. While the statistics of gastrostomy for acute troubles are encouraging, those of gastrostomy for chronic disorders affords but little prospect of success.

